# Cytotoxic Effects and Oxidative Stress Produced by a Cyanobacterial Cylindrospermopsin Producer Extract versus a Cylindrospermopsin Non-Producing Extract on the Neuroblastoma SH-SY5Y Cell Line

**DOI:** 10.3390/toxins15050320

**Published:** 2023-05-05

**Authors:** María G. Hinojosa, Antonio Cascajosa-Lira, Ana I. Prieto, Daniel Gutiérrez-Praena, Vitor Vasconcelos, Angeles Jos, Ana M. Cameán

**Affiliations:** 1 Area of Toxicología, Faculty of Pharmacy, Universidad de Sevilla, C/Profesor García González 2, 41012 Seville, Spainaclira@us.es (A.C.-L.);; 2 CIIMAR/CIMAR—Interdisciplinary Centre of Marine and Environmental Research, University of Porto, Terminal de Cruzeiros do Porto de Leixões, 4450-159 Matosinhos, Portugal; 3 Department of Biology, Faculty of Sciences, University of Porto, 4169-007 Porto, Portugal

**Keywords:** cylindrospermopsin, cyanobacterial extracts, cytotoxicity, oxidative stress, SH-SY5Y

## Abstract

The incidence and interest of cyanobacteria are increasing nowadays because they are able to produce some toxic secondary metabolites known as cyanotoxins. Among them, the presence of cylindrospermopsin (CYN) is especially relevant, as it seems to cause damage at different levels in the organisms: the nervous system being the one most recently reported. Usually, the effects of the cyanotoxins are studied, but not those exerted by cyanobacterial biomass. The aim of the present study was to assess the cytotoxicity and oxidative stress generation of one cyanobacterial extract of *R. raciborskii* non-containing CYN (CYN−), and compare its effects with those exerted by a cyanobacterial extract of *C. ovalisporum* containing CYN (CYN+) in the human neuroblastoma SH-SY5Y cell line. Moreover, the analytical characterization of potential cyanotoxins and their metabolites that are present in both extracts of these cultures was also carried out using Ultrahigh Performance Liquid Chromatography-Mass Spectrometry, in tandem (UHPLC-MS/MS). The results show a reduction of cell viability concentration- and time-dependently after 24 and 48 h of exposure with CYN+ being five times more toxic than CYN−. Furthermore, the reactive oxygen species (ROS) increased with time (0–24 h) and CYN concentration (0–1.11 µg/mL). However, this rise was only obtained after the highest concentrations and times of exposure to CYN−, while this extract also caused a decrease in reduced glutathione (GSH) levels, which might be an indication of the compensation of the oxidative stress response. This study is the first one performed in vitro comparing the effects of CYN+ and CYN−, which highlights the importance of studying toxic features in their natural scenario.

## 1. Introduction

Cyanobacteria are some of the most abundant microorganisms on Earth, capable of producing oxygen and converting CO_2_ into biomass using sunlight [1]. These photosynthetic prokaryotes grow to form blooms that are affected by temperature, sunlight, and the oxygen content of the environment where they proliferate, and thus, affect the biota in both aquatic and terrestrial ecosystems [2,3,4]. One of their most important features is the capacity of some strains to produce toxic secondary metabolites known as cyanotoxins [5,6]. Cylindrospermopsin (CYN) is one of the most studied cyanotoxins due to its emerging incidence, bioaccumulation, and multi-organ toxicity [7,8,9,10]. Moreover, up to 100% of total CYN can be released into water bodies, which likely increases absorption by aquatic organisms [11]. There are many species able to produce this toxin, such as *Raphidiopsis raciborskii, R. curvata, R. mediterranea, Anabaena lapponica, A. planctonica, Chrysosporum flos-aquae, C. gracile, C. ovalisporum, Dolichospermum mendotae, Lyngbya wollei, Umezakia natans* or *Oscillatoria* sp., among others [12,13,14]. Due to this, CYN has a cosmopolitan distribution pattern, as CYN-producer strains of cyanobacteria can be found in many aquatic environments. In addition, different environmental factors such as temperature, nutrient sources, and light intensity have been demonstrated to influence the CYN-production differently according to the producer organism [15]. 

Structurally, CYN is a tricyclic guanidine moiety combined with a hydroxymethyl uracil [16]. Chemically, this toxin is an alkaloid linked to a sulfated guanidinium zwitterion with a low molecular weight (415 Da), which makes it highly water-soluble and stable in a wide range of heat, light, and pH conditions [17]. The main route of CYN-exposure is the ingestion of contaminated water or food, or even accidental exposure during leisure activities [18,19,20]. This exposure is especially relevant in developing countries, where there is no proper water treatment [4]. In addition, CYN has demonstrated to be bioaccumulated through the trophic chain by aquatic organisms or vegetables irrigated with contaminated water, as the soil can also retain the toxin [21]. In this regard, CYN guideline values (GVs) for lifetime drinking water, short-term drinking water, and recreational water have been established in 0.7, 3 and 6 µg/L, respectively [22]. Moreover, it may be appropriate to consider reducing these GVs based on the relative exposure data for the population [23]. Nowadays, CYN concentrations greater than or equal to 1 μg/L have been detected in the 40.0%, 39.4%, 68.8%, 52.4%, 66.7% and 75% of water bodies in Europe, Asia, Oceania, North America, South America, and Africa, respectively, with up to 97 µg/L CYN being reported in finished drinking water [22]. This worldwide prevalence in water drinking reservoirs can imply a public health hazard, highlighting the ongoing need to understand and evaluate its toxic effects in a more realistic scenario [18]. The most important case of human intoxication due to CYN exposure was known as the “Palm Island Mystery Disease” [24]. Afterwards, epidemiological reports indicated that all of the patients had drunk water from a reservoir that presented a bloom of a CYN-producer strain of *R. raciborskii* with a high concentration of CYN [25]. 

The most described mechanisms of action for CYN are the protein and GSH synthesis inhibition [26,27,28]. In addition, CYN also enhances ROS production and oxidative stress [29], which could be involved in pathways for apoptosis or DNA damage [30]. Furthermore, CYN seems to be pro-genotoxic, needing a previous metabolic activation by the cytochrome P-450 complex [31]. One of the main targets for this alkaloid is the liver [23], although it has demonstrated to exert toxicity also in the lungs, heart, kidneys, marrow bone, thymus, gastrointestinal tract, adrenal glands, and the immune and nervous systems in several species [4] with this being the reason for its classification as a cytotoxin. However, the studies concerning its effects in the nervous system are still scarce, which is relevant due to the high incidence of degenerative diseases of the nervous system worldwide, and the possible causal contributions from environmental factors [32,33,34,35,36]. In this matter, some studies have been carried out using extracts of CYN-producing cyanobacteria, mainly in vivo [37,38,39,40], but the comparison between the toxic effects produced by a CYN-producing extract versus a CYN-non-producing extract in neuronal models has not been studied yet. In this sense, different CYN-producing and non-producing cyanobacterial species can coexist in nature, and the presence of other substances different from CYN can influence their toxicity. Thus, in order to elucidate the possible effects on the nervous system of cyanobacterial extracts, the aim of the present work was to study, for the first time, the cytotoxic effects and oxidative stress produced by a CYN-non-producing extract (*R. raciborskii*) (CYN−) and a CYN-producing extract (*C. ovalisporum*) (CYN+) on the human neuroblastoma SH-SY5Y cell line, and to stablish the correlation between the toxic effects induced by the CYN or other bioactive compounds present in the extracts. For this purpose, both extracts were previously characterized for the first time using UHPLC-MS/MS and an appropriate data processing by the available software (Compound Discoverer and Traze Finder) to determinate other toxins, metabolites, or bioactive molecules.

## 2. Results

### 2.1. Extracts Characterization

**Figure 1:**
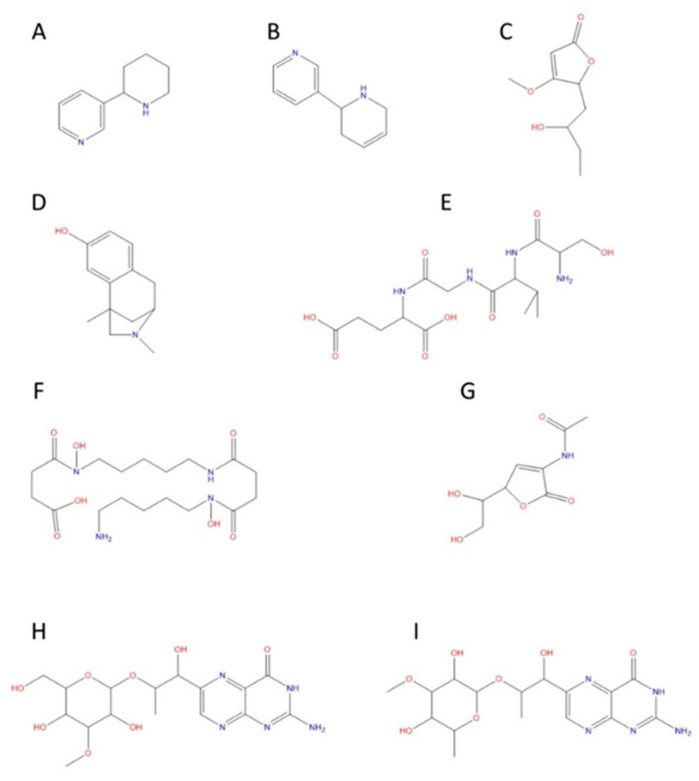
Molecular structures of the main compounds found in the extracts from a *R. raciborskii* and *C. ovalisporum* cultures. (**A**) Anabasine. (**B**) Anatabine. (**C**) Annularin G. (**D**) Aphanorphine. (**E**) AS-I Toxin. (**F**) Bisucaberin B. (**G**) Leptosphaerina. (**H**) Microcysbipterin. B (**I**) Microcysbipterin C.

**Figure 2:**
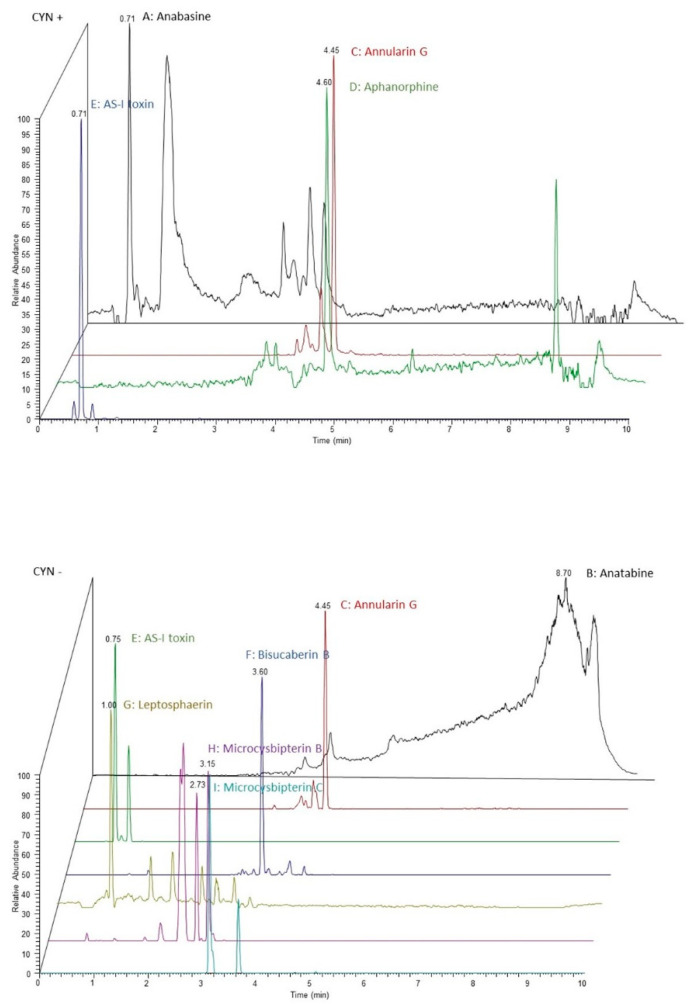
Chromatographic profile of CYN+ and CYN− extracts obtained after UHPLC-MS/MS determination. (A) Anabasine. (B) Anatabine. (C) Annularin G. (D) Aphanorphine. (E) AS-I Toxin. (F) Bisucaberin B. (G) Leptosphaerina. (H) Microcysbipterin B. (I) Microcysbipterin C. To improve visualization, each chromatogram is represented by a different color.

Once the bioactive compounds were extracted, they were analyzed by UHPLC-MS/MS and submitted to several databases related to metabolism and natural compounds (see Section 5.4). A total of 1596 compounds were found and identified in both extracts. The most outstanding compounds and whose presence have been corroborated in cyanobacteria are those present in Figure 1. The compounds found in the extract from *R. raciborskii* (CYN−) were: Anatabine, Annularin G, AS-I Toxin, Bisucaberin B, Leptosphaerina, Microcysbipterin B, and Microcysbipterin C., and from *C. ovalisporum* (CYN+) the following were found: Anabasine, Annularin G, Aphanorphine, and AS-I Toxin. Figure 2 shows the chromatographic profile of both extracts: CYN+ and CYN−.

In addition, numerous CYN metabolites have been identified in the extract from the *C. ovalisporum* culture, which are presented in Table 1. These metabolites could appear because of the following reactions: dehydration, reduction, oxidation, desaturation, nitro reduction, hydration, oxidative deamination to alcohol, sulfation, transformation of thiourea to urea, and acetylation.

### 2.2. Cytotoxicity Assays

**Figure 3:**
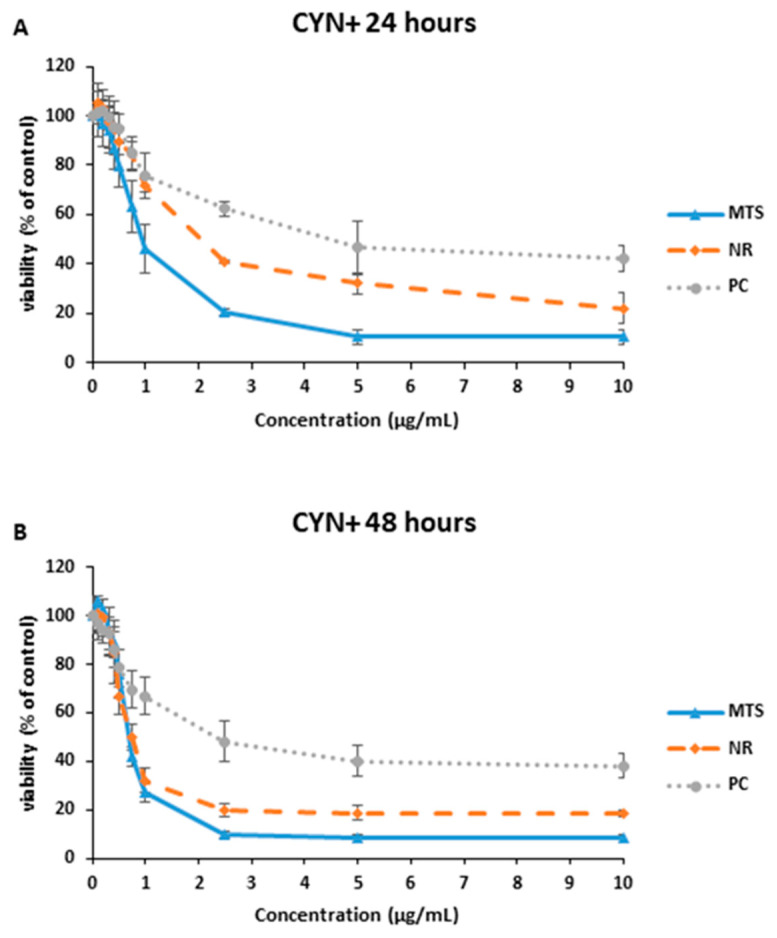
Reduction of tetrazolium salt (MTS), neutral red uptake (NR) and protein content (PC) on SH-SY5Y cells after 24 h (**A**) and 48 h (**B**) of exposure to 0–10 µg/mL CYN+. All values are expressed as mean ± s.d.

A concentration dependent decrease in the SH-SY5Y cell viability was observed after their exposure to 1–10 µg CYN/mL of CYN+ during 24 and 48 h for all the parameters measured (Figure 1). The EC_50_ values obtained after 24 h of exposure for the different assays were 1.111 ± 0.325, 2.085 ± 0.204 and 4.423 ± 0.330 µg CYN/mL for MTS, NR and PC, respectively (Figure 3A). Concerning the exposure for 48 h, CYN+ led to EC_50_ values of 0.691 ± 0.165, 0.733 ± 0.165 and 2.350 ± 0.506 µg CYN/mL for MTS, NR and PC, respectively (Figure 3B). At both exposure times, MTS resulted in the most sensitive biomarker, providing the lower EC_50_ values.

**Figure 4:**
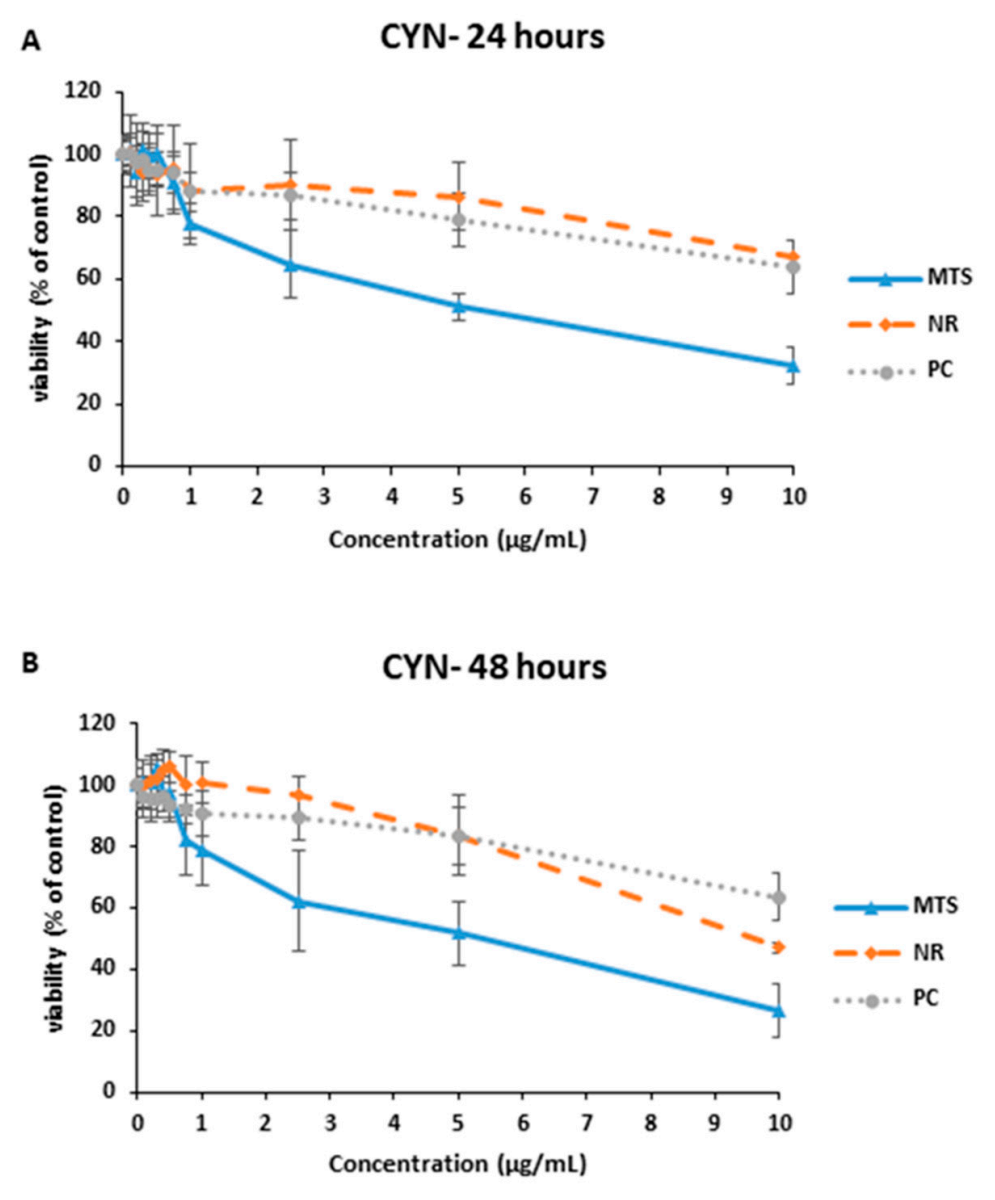
Reduction of tetrazolium salt (MTS), neutral red uptake (NR) and protein content (PC) on SH-SY5Y cells after 24 h (**A**) and 48 h (**B**) of exposure to the amount of CYN− equivalent to 0–10 µg/mL CYN+. All values are expressed as mean ± s.d.

In relation to the exposure to CYN− to the same amount of the extract than for CYN+, MTS provided EC_50_ values of 5.658 ± 1.180 and 5.164 ± 1.620 µg CYN+/mL equivalents after 24 and 48 h of exposure, respectively. The NR assay also led to a lower EC_50_ value after 48 h compared to the one after 24 h, being 9.669 ± 0.300 and >10 µg CYN+/mL equivalents, respectively. In addition, the PC assay led to EC_50_ values higher than 10 µg CYN+/mL equivalents at both exposure times (Figure 4). 

### 2.3. Oxidative Stress Assays

**Figure 5:**
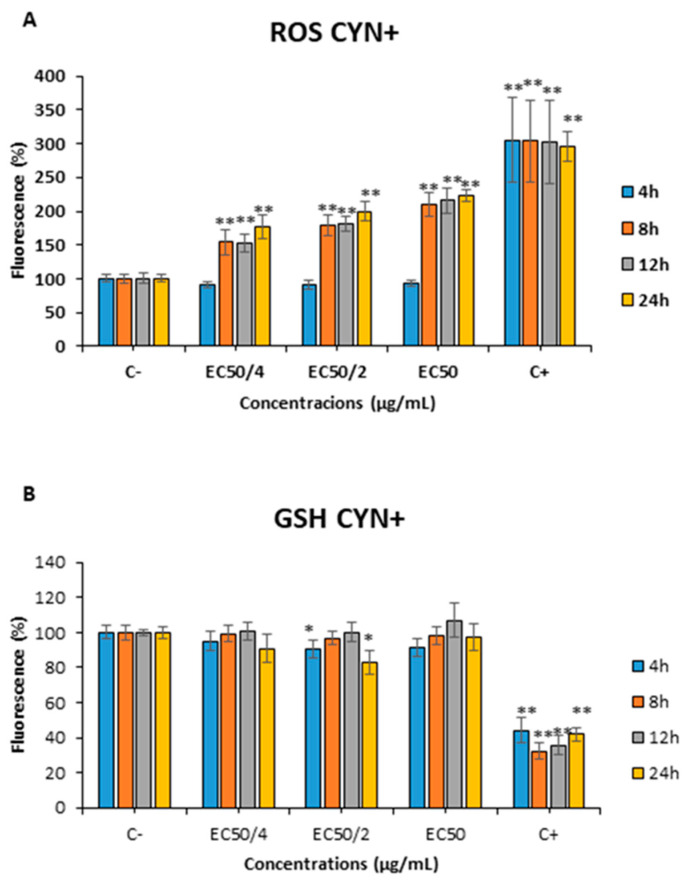
Reactive oxygen species (ROS) (**A**) and reduced glutathione (GSH) (**B**) levels on SH-SY5Y cells after 4, 8, 12 and 24 h of exposure to 0–1.1 µg/mL CYN+. All values are expressed as mean ± s.d. The significance levels observed are * *p* < 0.05 and ** *p* < 0.01 significantly different from control group.

The exposure to 0.275, 0.55 or 1.1 µg CYN/mL CYN+ (EC_50/4_, EC_50/2_ and EC_50_, respectively), led to significant changes in the ROS assay in SH-SY5Y cells after 8, 12 and 24 h (Figure 5A). However, ROS changes were not detected in this cell line after 4 h of exposure for any of the concentrations tested. Furthermore, a decrease in the GSH levels after exposure to 0.55 µg CYN/mL CYN+ after 4 and 24 h of exposure was also observed (Figure 5B).

**Figure 6:**
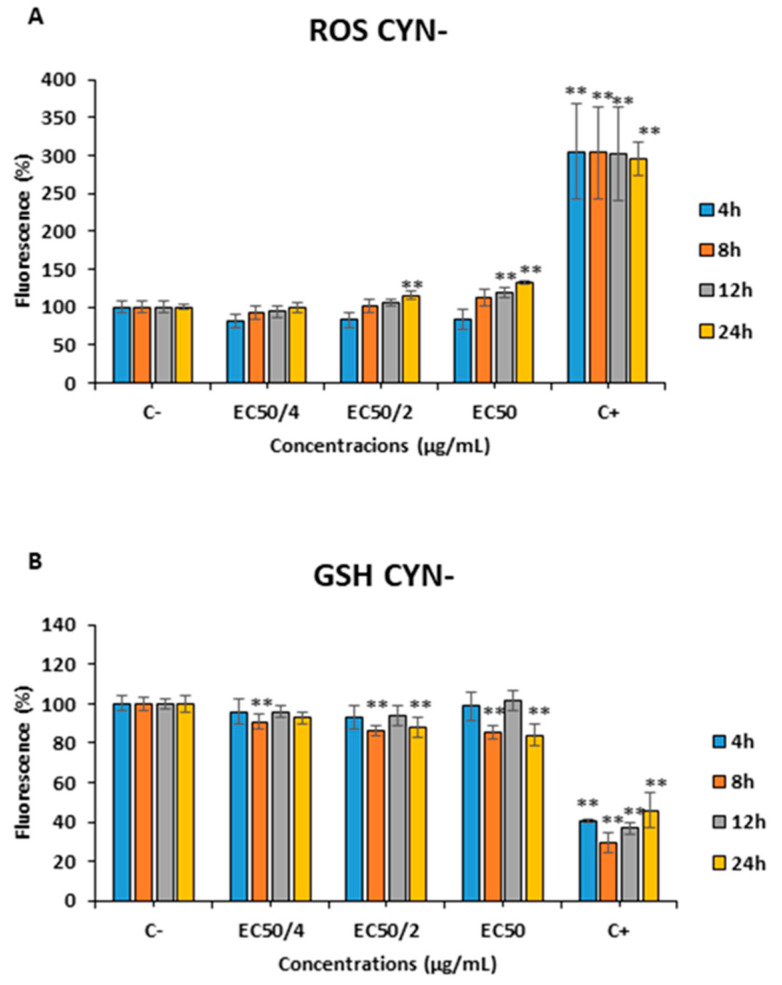
Reactive oxygen species (ROS) (**A**) and reduced glutathione (GSH) (**B**) levels on SH-SY5Y cells after 4, 8, 12 and 24 h of exposure to the amount of CYN− equivalent to 0–1.1 µg/mL CYN+. All values are expressed as mean ± s.d. ** Significantly different from control group (*p* < 0.01).

Concerning the non-producing extract, the same amount of CYN− caused an increase in the ROS levels after exposure to the highest concentrations only after the exposure for the longest periods (12 and 24 h) (Figure 6A). With respect to GSH levels, there was a significant decrease after the exposure to all the concentrations assayed after 8 and 24 h of exposure (Figure 6B).

## 3. Discussion

Cyanobacterial blooms have demonstrated to be able to cause toxicity to some extent in different experimental models as reviewed by Janssen [41]. Furthermore, it has also been reported that some cyanobacterial extracts containing cyanotoxins can cause different effects than the toxins themselves. In this sense, CYN is a cyanotoxin distributed worldwide due to the number of species able to produce this secondary metabolite. However, studies considering the effects of CYN− versus CYN+ are very scarce, while no studies in this regard have been reported in neuronal cells so far.

Taking all the above into account, cytotoxicity and oxidative stress assays were performed using a *R. raciborskii* CYN− strain and a *C. ovalisporum* CYN+ strain in the human neuroblastoma SH-SY5Y cell line. Concerning CYN−, the MTS assay led to EC_50_ values of 5.658 ± 1.180 and 5.164 ± 1.620 µg of CYN+/mL equivalents after 24 and 48 h of exposure, respectively, indicating cytotoxicity to some extent. This toxicity could be mainly produced by Anatabine: a major nicotinic receptor agonist [42], AS-I Toxin [43], Bisucaberin B: known cytostatic [44], and by Microcysbipterin B and C: protease inhibitors [45]. However, the same effects were observed after exposure to CYN+ at almost 5 times less concentration (EC_50_ values of 1.111 ± 0.325, and 0.691 ± 0.165 after 24 and 48 h, respectively), meaning that the effects observed after exposure to CYN+ could be mainly due to CYN and its different detected variants and not to the rest of the compounds present in the cyanobacterial extract, such as Anabasine: a major nicotinic receptor agonist [46], and AS-I Toxin [43], and both were detected in this extract. In addition, other potentially bioactive or toxic compounds could be present in the extracts and have been not identified in this work due to the conditions used in the CYN extraction. Moreover, the present study is limited by the inability to quantify minority metabolites detected in both extracts, which is due to the lack of commercially available standards. This obstacle is particularly prevalent when dealing with metabolites and compounds that have not yet been extensively described in scientific literature, as is the case in this particular research. However, the identification of such compounds can only be achieved through the use of reference spectra libraries. 

In regard to the CYN concentrations found in the environment, they can vary considerably based on multiple factors [4]. Up to 1050 μg/L CYN has been detected in an Australian water supply [11]. Taking into account that up to 90% of the total CYN in surface water is in the dissolved fraction, rather than intracellular [22], the concentrations used in this study (0–10 µg/mL) reflect actual exposure scenarios in the environment.

In order to compare the effects observed after exposure to CYN+, Hinojosa et al., [35] in the same experimental model, obtained an EC_50_ value of 0.87 ± 0.13 and 0.32 ± 0.08 µg/mL after the same exposure times, but used pure CYN [35,47]. Thus, CYN seems to be a bit less toxic in the extract than if it were isolated, probably due to some interaction between the rest of the compounds of the extract with the toxin, highlighting the importance of considering other substances that may be present in natural conditions. The only study performed in vitro with neuronal models exposed to cyanobacterial extracts, reported no alterations in neurons of *Helix pomatia* that were acutely exposed to crude extracts of *R. raciborskii* containing CYN [48]. However, the CYN-concentration was not indicated. Concerning the effects of pure CYN in cytotoxicity in neuronal cell models, Takser et al. [49] also obtained a concentration-dependent effect after exposure to 0.042 and 4.2 µg/mL in N2a murine neuroblastoma derived cells and BV-2 microglia murine cells with N2a cells being more sensitive to CYN than BV-2 cells, which highlights the importance of the experimental model. Furthermore, the effects of pure CYN have been investigated in terms of synapsis effects on murine primary neurons [36], and pure CYN led to a decrease on cell viability at the same concentrations than those tested in the present study, together with a decrease on the number of synapsis at similar concentrations than EC_50_ and EC_50/2_ in our study [36]. 

Regarding the comparison between cells exposed to CYN+ and pure CYN in non-nervous cellular models, different results have been described in scientific literature. Contrary to the results obtained in our study, Gutierrez–Praena et al. [50] performed the comparison between CYN+ and pure CYN in the human liver cancer cell line HepG2, obtaining higher toxicity exerted by CYN+. These differences obtained in the CYN– extracts from *R. raciiborski* could be mainly due to the experimental model used (SH-SY5Y vs. HepG2 cell lines). In addition, even though the CYN- species is the same, the cultivation conditions (growth time, biomass concentration, etc.) and extraction process could cause variations in the composition of bioactive substances other than cyanotoxins in cyanobacterial CYN- extracts. However, in the same experimental model, several authors obtained different responses. In this sense, Bain et al. [51] obtained significant changes from 1 µg/mL after exposure for 24, 48, and 72 h to pure CYN, while Neumann et al. [52] demonstrated again that the extract produced a lower response than the one obtained by the pure toxin, in agreement with our data. 

In a different cell line, Bain et al. [51] performed the MTS assay in Caco-2 cells using pure CYN, detecting a significant reduction of cell viability after 24 and 48 h of exposure from 2.5 µg/mL, while after 72 h, the changes were significant from 1 µg/mL. However, when Gutierrez–Praena et al. [30] performed the same experiment, also using pure CYN, they reported a significant decrease in cell viability at lower concentrations. In this regard, according to Neumann et al. [52], CYN+ from *R. raciborskii* was demonstrated to be less toxic after 24 h compared to the results obtained by pure CYN, which agrees with our results by leading to significant changes from 2.5 µg CYN/mL CYN+, while after 48 h these changes started to be significant from 0.25 µg CYN/mL. 

Furthermore, in the present study, a significant increase in ROS levels was detected after all the concentrations of exposure assayed for 8, 12, and 24 h in the cells exposed to the CYN+ extract. However, almost no significant changes were observed in GSH levels, which could indicate an ineffective response against ROS levels. Nonetheless, CYN− extracts led to the opposite by increasing ROS levels only after the highest concentrations and times of exposure, but caused a significant reduction of GSH levels after all the concentrations of exposure at different time points. This fact could indicate that the oxidative stress produced by the extracts might not be due just to the presence of CYN but also the presence of some other compounds, such as Anatabine, AS-I Toxin, Bisucaberin B, Microcysbipterin B and C, even though the oxidizing activity of these substances has not been described yet in the literature.

In a similar way, other authors have also found increased ROS levels in HepG2 cells with CYN+ and pure CYN [53,54]. Concerning CYN−, there are no previous in vitro studies performed with these extracts in this regard, in any experimental model, to be able to compare our results. 

Specifically, in terms of neurotoxicity, in vivo studies after exposure to CYN cyanobacterial extracts have been performed in aquatic animals. In this regard, Kinnear et al. and White et al., [37,38] reported histopathological changes in the encephalon and behavioral changes, respectively, after seven-days exposure to whole cell extracts and live cell cultures of *R. raciborskii* in *Bufo marinus* tadpoles. Furthermore, Guzmán–Guillén et al. [39] exposed tilapia fish to a culture of *C. ovalisporum* containing CYN for 14 days, which led to the inhibition of acetylcholinesterase activity, an increase in lipid peroxidation, and serious histopathological damage. In addition, these authors detected CYN in all brain samples, which agrees with the results obtained in Da Silva et al. [40], who exposed *Hoplias malabaricus* to both purified CYN and an extract of *R. raciborskii* containing CYN for 7 and 14 days by intraperitoneal injection. In this study, the authors also reported an increase on the acetylcholinesterase activity after 7 days of exposure to CYN+, and a decrease after 14 days of exposure to CYN. Furthermore, these authors also detected an increase in oxidative stress by an increase in glutathione-S-transferase activity (GST), and lipid peroxidation after both 7 and 14 days of exposure to both forms of CYN (CYN+ and pure CYN). Thus, these authors reported differences between the effects produced by CYN+ and CYN itself. 

In general, these facts highlight that the importance of studying the cyanobacterial extracts, as well as their own toxicity, cannot be ignored. In addition, the presence of these bioactive compounds may play an important role that should be taken into account in order to perform a more rigorous evaluation of the toxic implications of cyanobacterial blooms in different organisms, and in this case, the possible neurotoxic properties of several compounds that may be present in the cyanobacterial extracts.

## 4. Conclusions

For the first time, the present study showed the cytotoxic effects which are caused by the exposure to cyanobacterial extracts in the neuronal SH-SY5Y cell line, resulting in more toxicity in CYN+ compared to CYN−. In addition, the characterization of both extracts (UHPLC-MS/MS) revealed the presence of different substances and products derived from CYN with neurotoxic potential in these culture extracts, such as Anatabine, AS-I Toxin, Bisucaberin B, Anabasine, Microcysbipterin B and C. Furthermore, our results demonstrated that the CYN+ extract caused an increase in ROS levels without significant alterations of the GSH levels. On the other hand, the CYN− extract induced an increment in ROS levels only at the highest concentrations and times of exposure assayed, but a significant decrease in GSH levels at all the concentrations assayed, which could be explained by the presence of other compounds able to induce oxidative stress in the neuronal cells. In general, comparing the effects produced in both culture extracts (CYN+ vs. CYN−), the greatest toxicity seems to be due to the presence of CYN when compared to the other detected compounds. Thus, these results highlight the importance of studying not only the isolated cyanotoxin but also in their natural environment, as well as no toxins producing cyanobacterial cultures to test more realistic exposure scenarios.

## 5. Materials and Methods

### 5.1. Supplies and Chemicals

Gibco (Biomol, Sevilla, Spain) has provided the fetal bovine serum (FBS), minimum essential medium (MEM) and cell culture reagents. HPLC-grade methanol, dichloromethane, trifluoroacetic acid (TFA) and formic acid were obtained from Merck (Darmstadt, Germany). Deionized water (>18 MΩcm-1 resistivity) was obtained from a Milli-Q water purification system (Millipore, Bedford, MA, USA). The MTS (3-(4,5- dimethylthiazol-2-yl)-5-(3-carboxymethoxyphenyl)-2-(4-sulphophenyl)-2H-tetrazolium salt) Cell Titer 96^®^ AQueous One Solution Cell Proliferation Assay was purchased in Promega (Biotech Iberica, Madrid, Spain). The neutral red (NR) and the Bradford reagent were obtained from Sigma-Aldrich (Madrid, Spain). BOND ELUT^®^ Carbon cartridges (500 mg, 6 mL) were supplied by Agilent Technologies (Amstelveen, The Netherlands, Europe). CYN (95%), (+/−) ATX, MC-LR, MC-RR and MC-YR (99%) standards were supplied by Enzo Life Sciences (Lausen, Switzerland).

### 5.2. Model System

Human neuroblastoma SH-SY5Y cells were obtained from the American Type Culture Collection (CRL-2266). This cell line was maintained in an MEM and F-12 (1:1) medium supplemented with 10% FBS, 1% L-glutamine 200 mM, 1% sodium pyruvate, 1% non-essential amino acids, and 1% penicillin/streptomycin solution, in an atmosphere containing 5% CO_2_ at 95% relative humidity at 37 °C (CO_2_ incubator, NuAire^®^, Madrid, Spain). Cells were grown to 80% of confluence in 75 cm^2^ plastic flasks. Cells were harvested with 0.25% trypsin-EDTA (1X) twice per week. The quantification of the cells was performed in a Neubauer chamber. SH-SY5Y cells were plated at a density of 1 × 10^5^ cells/mL to perform all the experiments. 

The SH-SY5Y cell line has been widely used in preliminary neurotoxicity studies because it has important advantages [55]. There are also several publications that use this cellular model to measure GSH content and show it to be a suitable model for this purpose [56,57].

### 5.3. Chrysosporum Ovalisporum and Raphidiopsis Raciborskii Cultures

*C. ovalisporum* (LEGE X-001) cyanobacterial CYN-producing strain (CYN+) was originally isolated from Lake Kinneret [58] and kindly supplied by the laboratory of Dr. Vitor Vasconcelos from the Marine Research Center—CIIMAR (Porto, Portugal) and *R. raciborskii* (LEGE 99044) cyanobacteria CYN-non-producing strain (CYN−) from the LEGE culture collection with the culture conditions described in Guzmán–Guillén et al. [59]. 

### 5.4. CYN Determination and Characterization of Cultures

CYN extraction from the lyophilized cultures (*C. ovalisporum* CYN+ and *R. raciborskii* CYN−) was performed according to the validated method published in Guzmán–Guillén et al. [59]. 

**Figure 7:**
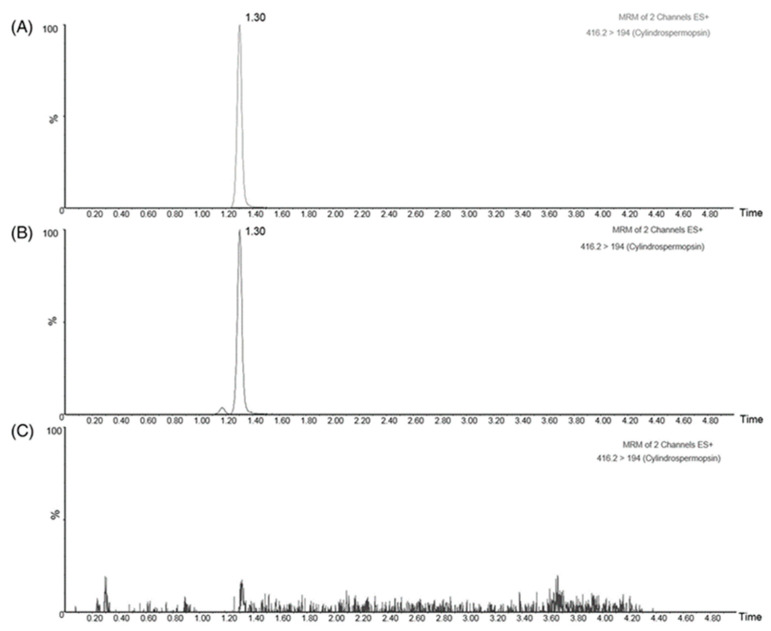
MRM chromatograms by UPLC-MS/MS of (**A**) a CYN standard solution (100 μg/L); (**B**) a diluted CYN-producing (CYN+) extract from a *C. ovalisporum* culture, and (**C**) a non-CYN producing extract (CYN−) from a *R. raciborskii* culture.

CYN was only detected in the CYN+ extract with a retention time of 1.31 min, obtaining 91.50 μg CYN/mL concentration in the CYN-producing extract. No presence of CYN was found in the non-CYN-producing extract (Figure 7). 

Moreover, both extracts were analyzed for the presence of other cyanotoxins or metabolites using analytical techniques previously used in our laboratory [60]. For this purpose, standards of the following cyanotoxins were used: Anatoxin A, Microcystin-LR, -RR and -YR. Finally, a metabolomic study was carried out to search for molecules derived from the toxins and bioactive compounds using the Compound discoverer 3.2 software (Thermo Fisher Scientific, Madrid, Spain) and the databases: BioCyc, Food and agricultural organization, FooDB, Human Metabolome database, KEGG, mass bank, Nature chemistry, Phenol-explorer and Plant-Cyc.

### 5.5. Cytotoxicity Assays

SH-SY5Y cells were seeded out for basal toxicity tests in 96 well tissue culture plates, and after being incubated at 37 °C for 24 h, they were exposed to the extracts. To select the amount of extract for exposure, the producer extract was used as a reference. Thus, once CYN was quantified in this extract (CYN+), the required amounts of extract containing CYN concentrations in the range of 0–10 µg CYN/mL of this toxin were calculated. The same amount of CYN− extract was used to perform the cytotoxicity assays. After this time, the medium was replaced with the exposure solutions in the plates and then they were incubated for 24 and 48 h at 37 °C at 5% CO_2_. The basal cytotoxicity endpoints assayed were tetrazolium salt reduction (MTS), supravital dye neutral red cellular uptake (NR) and protein content (PC). All the assays present in this work were performed by triplicate. 

MTS reduction was measured according to the procedure described in Barltrop et al. [61]. After the addition of the MTS compound to the wells and the incubation during 3 h in darkness, the absorbance was immediately measured at 490 nm.

Neutral red uptake was performed according to the procedure indicated in Borenfreund and Puerner [62]. The NR absorbed by the cells was quantified at 540 nm.

PC was quantified following the method reported in Bradford [63] with modifications [64] and read the absorbance at 620 nm. 

### 5.6. Oxidative Stress Assays

#### 5.6.1. Reactive Oxygen Species (ROS) Generation

The ROS production was assessed by using the dichlorofluorescein (DCF) assay in 96 well plates according to Puerto et al. [65] with some modifications [35,48,66,67]. 

#### 5.6.2. Glutathione Content (GSH)

Glutathione (GSH) content was evaluated by reaction with the fluorescent probe monochlorobimane (mBCL). Cells were exposed to the extracts at the same concentrations as the ones used in the ROS generation assay and then incubated for 4, 8, 12, or 24 h. The GSH synthesis inhibitor, buthionine sulfoximine (BSO, 1 mM), was used as the positive control. After the exposure, cells were incubated for 20 min at 37 °C in the presence of 40 µM mBCL. Later, cells were washed with PBS and the fluorescence measured at 380 nm (excitation) and 460 nm (emission).

### 5.7. Calculations and Statistical Analysis

The data were presented as mean ± standard deviation (SD) in relation to control. Statistical analysis was carried out using analysis of variance (ANOVA), followed by Dunnett’s multiple comparison tests using GraphPad InStat software (GraphPad Software Inc., La Jolla, CA, USA). Differences were considered significant from *p* < 0.05. EC_50_ and the values were derived by linear regression in the concentration response curves.

## Figures and Tables

**Table 1:** Summary of metabolites found in *C. ovalisporum* extract (CYN+) by UPLC–MS/MS.

Formula	Parent Compound	Transformations	Composition Change	Delta Mass [ppm]	*m*/*z*	Retention Time [min]	Area Max
C_15_ H_21_ N_5_ O_6_ S	cylindrospermopsin	Dehydration, Reduction	-(O)	0.15	399.12131	3.097	1.48 × 10^8^
C_15_ H_21_ N_5_ O_6_ S	cylindrospermopsin	Desaturation, Nitro Reduction	-(O)	0.15	399.12131	3.097	1.48 × 10^8^
C_15_ H_20_ N_4_ O_6_	cylindrospermopsin	Hydration, Oxidative Deamination to Alcohol	-(H N O S)	0.01	352.13829	2.878	5.76 × 10^6^
C_15_ H_23_ N_5_ O_3_	cylindrospermopsin	Hydration, Nitro Reduction	-(O_4_ S) + (H_2_)	0.4	321.18022	3.073	3.87 × 10^7^
C_15_ H_19_ N_5_ O_7_ S	cylindrospermopsin	Desaturation	-(H_2_)	0.72	413.10082	2.821	1.91 × 10^7^
C_15_ H_19_ N_5_ O_7_ S	cylindrospermopsin	Dehydration	-(H_2_)	0.72	413.10082	2.821	1.91 × 10^7^
C_15_ H_19_ N_5_ O_7_ S	cylindrospermopsin	Sulfation	-(H_2_)	0.72	413.10082	2.821	1.91 × 10^7^
C_17_ H_23_ N_5_ O_9_	cylindrospermopsin	Thiourea to Urea, Acetylation	-(S) + (C_2_ H_2_ O_2_)	−0.17	441.1495	3.039	1.40 × 10^7^
C_15_ H_21_ N_5_ O_5_	cylindrospermopsin	Dehydration, Nitro Reduction, Thiourea to Urea	-(O_2_ S)	−0.39	351.15413	1.102	1.25 × 10^7^
C_15_ H_21_ N_5_ O_5_	cylindrospermopsin	Hydration	-(O_2_ S)	−0.39	351.15413	1.102	1.25 × 10^7^
C_15_ H_21_ N_5_ O_8_ S	cylindrospermopsin	Oxidation	+(O)	−0.6	431.11083	1.726	1.14 × 10^7^
C_15_ H_21_ N_5_ O_8_ S	cylindrospermopsin	Hydration, Sulfation	+(O)	−0.6	431.11083	1.726	1.14 × 10^7^
C_15_ H_21_ N_5_ O_8_ S	cylindrospermopsin	Desaturation	+(O)	−0.6	431.11083	1.726	1.14 × 10^7^
C_15_ H_21_ N_5_ O_5_	cylindrospermopsin	Dehydration, Nitro Reduction, Thiourea to Urea	-(O_2_ S)	0.3	351.15437	2.684	1.06 × 10^7^
C_15_ H_21_ N_5_ O_5_	cylindrospermopsin	Hydration	-(O_2_ S)	0.3	351.15437	2.684	1.06 × 10^7^
C_15_ H_17_ N_5_ O_3_	cylindrospermopsin	Dehydration	-(H_4_ O_4_ S)	0.47	315.13329	3.853	9.31 × 10^6^
C_15_ H_19_ N_5_ O_9_	cylindrospermopsin	Desaturation, Oxidation, Thiourea to Urea	-(H_2_ S) + (O_2_)	−0.68	413.118	1.25	8.46 × 10^6^
C_15_ H_19_ N_5_ O_9_	cylindrospermopsin	Desaturation, Thiourea to Urea	-(H_2_ S) + (O_2_)	−0.68	413.118	1.25	8.46 × 10^6^
C_15_ H_21_ N_5_ O_4_	cylindrospermopsin	Reduction	-(O_3_ S)	−1.11	335.15898	2.363	6.12 × 10^6^
C_15_ H_19_ N_5_ O_8_ S	cylindrospermopsin	Desaturation, Oxidation	-(H_2_) + (O)	−0.33	429.09529	1.368	6.09 × 10^6^
C_15_ H_19_ N_5_ O_8_ S	cylindrospermopsin	Desaturation	-(H_2_) + (O)	−0.33	429.09529	1.368	6.09 × 10^6^
C_15_ H_19_ N_5_ O_8_ S	cylindrospermopsin	Oxidation, Sulfation	-(H_2_) + (O)	−0.33	429.09529	1.368	6.09 × 10^6^
C_15_ H_19_ N_5_ O_8_ S	cylindrospermopsin	Desaturation, Desaturation	-(H_2_) + (O)	−0.33	429.09529	1.368	6.09 × 10^6^
C_15_ H_23_ N_5_ O_10_	cylindrospermopsin	Hydration, Oxidation, Thiourea to Urea	-(S) + (H_2_ O_3_)	−0.57	433.14424	0.92	5.50 × 10^6^
C_15_ H_23_ N_5_ O_10_	cylindrospermopsin	Hydration, Thiourea to Urea	-(S) + (H_2_ O_3_)	−0.57	433.14424	0.92	5.50 × 10^6^
C_15_ H_23_ N_5_ O_10_	cylindrospermopsin	Oxidation, Thiourea to Urea	-(S) + (H_2_ O_3_)	−0.57	433.14424	0.92	5.50 × 10^6^
C_15_ H_19_ N_5_ O_3_ S	cylindrospermopsin	Dehydration, Dehydration, Nitro Reduction	-(H_2_ O_4_)	−0.68	349.12062	1.598	4.34 × 10^6^
C_15_ H_21_ N_5_ O_3_	cylindrospermopsin	Nitro Reduction, Oxidation	-(O_4_ S)	0.21	319.16451	3.245	3.99 × 10^6^
C_15_ H_23_ N_5_ O_9_	cylindrospermopsin	Hydration, Thiourea to Urea	-(S) + (H_2_ O_2_)	−0.86	417.14922	1.732	3.90 × 10^6^
C_15_ H_23_ N_5_ O_9_	cylindrospermopsin	Reduction, Thiourea to Urea	-(S) + (H_2_ O_2_)	−0.86	417.14922	1.732	3.90 × 10^6^
C_15_ H_23_ N_5_ O_9_	cylindrospermopsin	Thiourea to Urea	-(S) + (H_2_ O_2_)	−0.86	417.14922	1.732	3.90 × 10^6^
C_15_ H_21_ N_5_ O_9_ S	cylindrospermopsin	Oxidation, Oxidation	+(O_2_)	−1.28	447.10542	0.957	3.10 × 10^6^
C_15_ H_21_ N_5_ O_9_ S	cylindrospermopsin	Oxidation	+(O_2_)	−1.28	447.10542	0.957	3.10 × 10^6^
C_15_ H_21_ N_5_ O_9_ S	cylindrospermopsin	Desaturation, Oxidation	+(O_2_)	−1.28	447.10542	0.957	3.10 × 10^6^
C_15_ H_25_ N_5_ O_9_	cylindrospermopsin	Hydration, Reduction, Thiourea to Urea	-(S) + (H_4_ O_2_)	−0.41	419.16506	0.912	2.84 × 10^6^
C_15_ H_25_ N_5_ O_9_	cylindrospermopsin	Reduction, Thiourea to Urea	-(S) + (H_4_ O_2_)	−0.41	419.16506	0.912	2.84 × 10^6^
C_17_ H_20_ N_4_ O_6_	cylindrospermopsin	Oxidative Deamination to Alcohol, Acetylation	-(H N O S) + (C_2_)	0.02	376.13829	4.091	2.74 × 10^6^
C_15_ H_17_ N_5_ O_3_	cylindrospermopsin	Dehydration	-(H_4_ O_4_ S)	1.33	315.13356	5.265	2.39 × 10^6^
C_15_ H_25_ N_5_ O_9_ S_2_	cylindrospermopsin	Nitro Reduction, Sulfation	+(H_4_ O_2_ S)	−0.71	483.10903	0.93	2.10 × 10^6^

## Data Availability

Not applicable.
